# In Situ Remediation of Phosphogypsum with Water-Washing Pre-Treatment Using Cemented Paste Backfill: Rheology Behavior and Damage Evolution

**DOI:** 10.3390/ma14226993

**Published:** 2021-11-18

**Authors:** Yikai Liu, Qiusong Chen, Yunmin Wang, Qinli Zhang, Hongpeng Li, Chaoyu Jiang, Chongchong Qi

**Affiliations:** 1 Department of Geosciences, University of Padova, 35131 Padova, Italy; yikai.liu@phd.unipd.it; 2 Sinosteel Maanshan General Institute of Mining Research Co., Ltd., Maanshan 243000, China; Wangyunmin@csu.edu.cn; 3 School of Resources and Safety Engineering, Central South University, Changsha 410083, China; zhangqinlicn@126.com (Q.Z.); 13278883353@163.com (C.J.); chongchong.qi@gamil.com (C.Q.); 4 Yinshan Mining Co., Ltd., Jiangxi Copper Group, Dexing 334200, China; lihongpeng88@126.com

**Keywords:** cemented paste backfill, waste recycle, phosphogypsum, unconfined compressive strength, mechanical properties

## Abstract

The accumulation of original phosphogypsum (OPG) has occupied considerable land resources, which have induced significant environmental problems worldwide. The OPG-based cemented paste backfill (OCPB) has been introduced as a promising solution. In this study, a water-washing pre-treatment was used to purify OPG, aiming to optimize the transport performance and mechanical properties of backfills. The overall results proved that in treated phosphogypsum-based cemented paste backfill (TCPB), the altered particle size distribution can alleviate the shear-thinning characteristic. The mechanical properties were significantly optimized, of which a maximum increase of 183% of stress value was observed. With more pronounced AE signals, the TCPB samples demonstrated better residual structures after the ultimate strength values but with more unstable cracks with high amplitude generated during loading. Principal component analysis confirmed the adverse effects of fluorine and phosphorus on the damage fractal dimensions. The most voluminous hydration products observed were amorphous CSH and ettringite. The interlocked stellate clusters may be associated with the residual structure and the after-peak AE events evident in TCPB, indicate that more significant stress should be applied to break the closely interlocked stitches. Ultimately, the essential findings in this experimental work can provide a scientific reference for efficient OPG recycling.

## 1. Introduction

Phosphate plays an indispensable role in modern agriculture, owing to the rapidly growing demand for phosphorus fertilizer [1,2,3]. However, the antiquated phosphate rock excavation and beneficiation technology can result in prolific problems, for instance, the forming of goaf [4,5,6], the consumption of land resources [7,8], and the leaching of hazardous substances [9,10]. What is more, as a typical by-product generated from the phosphate fertilizer production procedures, the industrial recycling of original phosphogypsum (OPG) has been strongly constrained by its physical properties and chemical composition [11]. Accordingly, the stockpiles of OPG pose a significant threat to natural aquifers and soil, especially for groundwater, which can be used as drinking water [11,12]. According to previous statistics, the annual storage of OPG is predicted to exceed 200 million tonnes in the next decade [13]. Since these hazards have increasingly raised serious concerns, scientific developments regarding efficient and eco-friendly solutions have been continually progressing, for instance, circular agricultural production [14,15], rare earth element recovery [16], and building material manufacture [17,18,19,20,21]. However, the successful commercialization of recycling OPG is far from an apparent breakthrough [22].

Cemented paste backfill (CPB), as a relatively safe and reliable approach to broaden the mining solid waste and benefit by-product recycling [23,24,25], has been introduced into the recovery of OPG [22,26]. According to current studies, OPG lacks sufficient mechanical property and durability to be used independently [27,28,29]. Besides, the impurities (mainly phosphorus and fluoride) reported can deteriorate the workability and mechanical properties of OPG based CPB (OCPB) [1,11,30]. The application of various OPG pre-treatments could improve the properties of OPG-binder systems, for instance, calcination [31], water washing [32], and membrane technology [33]. However, it has to be mentioned that using complicated pre-treatment systems in engineering applications can further squeeze the unprofitable margins. As a widely used purification method [34,35], a water-washing was found that can effectively optimize the particle size distribution (PSD) and reduce the soluble phosphorus and fluoride content of OPG [32,36]. Although many works have already identified the mechanical performance and environmental feasibility of OCPB, researchers have seldom investigated the rheology characteristics and acoustic emission (AE) signals which are also essential factors for converting OCPB from lab scale to industrial application. The precise knowledge of rheological behavior, which reflects the cohesion and frictional resistance of the pastes, is of great importance to advance the backfill procedures and enhance production efficiency. Meanwhile, as a widely recognized failure detection method, acoustic emission (AE) testing technology is commonly used in investigating and monitoring rock damage assessment [37], fracture classification [37,38,39,40], and landslide warning [41]. Although a positive correlation between the AE signals and the strain–stress curve was found, the role of different phases in CPB in AE testing has not always been evident and apparent [42,43].

**Figure 1:**
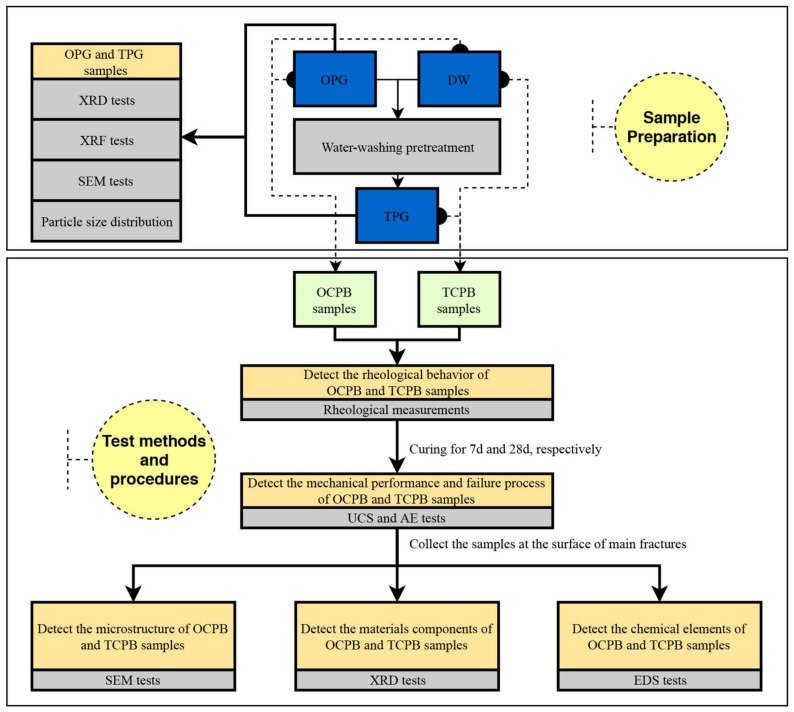
Flowchart for this study.

For this reason, the main objective of this study has been to analyze the influence of water-washing pre-treatment on the rheological behavior and AE parameters of OCPB and TPCB samples, emphasizing the monitoring of the complex fracture behavior after hardening. The TPG was prepared through deionized water (DW) washing, and OCPB and TCPB samples with two mix proportions were accordingly prepared for rheology behavior, mechanical properties, AE characteristics, mineralogical characterization, and microstructures examination. This study aims to provide a laboratory basis for early alarm mechanical failures when applying OPG to underground structures. A flow chart is plotted in Figure 1.

## 2. Sample Preparation and Test Methods

### 2.1. Materials

The OPC used in this study was CEM I 42.5R, with a specific surface area of 1.69 m^2^/g. OPG was sampled from a phosphoric acid production plant in Hubei province, China. The TPG was obtained by previous water-washing pre-treatment [32]. Before the mixture manufacture and characterization experiments, OPG and TPG were both dried at 45 ± 5 °C until constant weight [32,44,45].

### 2.2. Mix Proportions

The OCPB and TCPB were prepared according to Table 1. After being stirred homogeneously, the mixtures were cast into plastic, cylindrical molds (Ø50 × 100 mm) and hydrated for 24 h. Subsequently, the OCPB and TCPB samples were transferred into the chamber with a controlled temperature of 25 ± 2 °C and humidity of 90 ± 5% [46]. After being cured for different periods, the hardened samples were extracted from the plastic molds and then subjected to the following test procedures. Taking into account the field conditions, all the samples were not sealed. As mixing water strongly affects CPB’s hydration mechanisms [1], deionized water (Solar-bio Co. Ltd., Beijing, China) was provided in this study.

### 2.3. Test Methods

#### 2.3.1. Characterization Experiments

The chemical compositions and particle size distribution of the OPG and TPG samples were determined by X-ray fluorescence (XRF, Bruker S4 Pioneer XRF analyzer, Karlsruhe, German) and a laser particle analyzer (Master Sizer 2000, Worcester, UK). The OCPB and TCPB used for the following analysis were collected at the surface of the main fractures in each loaded sample, then dried at 50 ± 5 °C for 24 h and powered in an agate mortar. XRD analysis of the collected pieces was performed using an X-ray diffractometer (D8 Advance, Bruker, Karlsruhe, Germany) employed with Cu Kα line radiation. The XRD patterns were appraised using the references in the PDF-2 database (PDF-2 International Centre for Diffraction Data, Newtown Square, PA, USA). SEM/EDX observation was employed for the microstructure of backfills on the JSM-6490LV scanning electron microscope (JEOL Ltd., Tokyo, Japan). Before being subjected to the SEM/EDX tests, the powder samples were gilded to confirm good conductivity.

#### 2.3.2. Experiments of Rheological Characteristics

Rheological measurements were carried out using a controlled stress rheometer equipped with a coaxial rotating cylinder (Viscotester 550 Rotational Viscometer, Haake Technik GmbH, Vreden, Germany). The prepared OCPB and TCPB samples were first mixed for 2 min to guarantee the homogeneity of the mortars. Then, before being poured into the rheometer, the mortars were kept at rest for 3 min. During the equilibrium test, the shear rate was ramped up from 0 to 45 s^−1^ in 180 s. All rheological measurements were performed at a relatively steady ambient temperature of 20 ± 1 °C.

#### 2.3.3. Experiments of Mechanical Properties and AE

Considering the poor mechanical performance of 7 day cured OCPB samples, the specimens were subjected to a biomechanical testing machine (23 MTS Insight, produced by MTS Systems Co., Ltd., Eden Prairie, MN, USA, with controlled loading speed: 0.1 mm/min, loading capability: 30 kN) [47] adopted with the PCI-2 AE testing system (Physical Acoustic Corporation, United States, preamplification: 40 dB, threshold value: 40 dB, probe resonance frequency: 20–100 kHz). The schematic diagram of UCS and AE tests is shown in Figure S1. Besides, Vaseline and adhesive tape were used to couple the contact surface of the AE probe and OCPB to ensure signal reception [48]. To simplify the data set processing, UCS and AE measurement systems must be kept synchronized to record interchangeable time parameters.

## 3. Results and Discussion

### 3.1. Raw Materials Characterization

**Figure 2:**
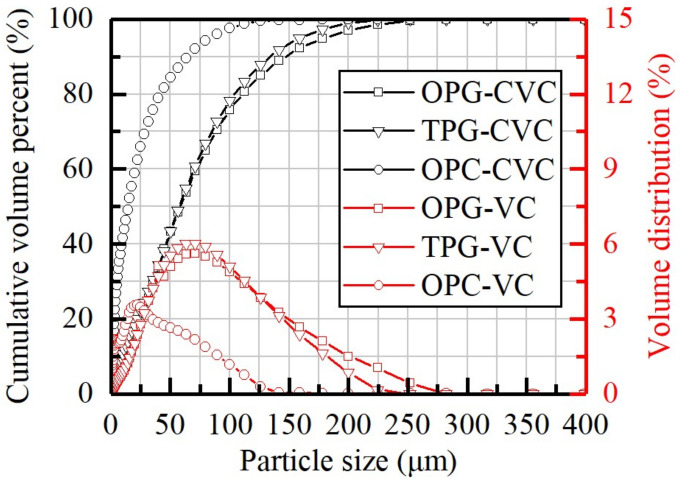
The particle size distribution of the raw materials. CVC: cumulative volume percentage curves; VC: volume distribution curves.

**Figure 3:**
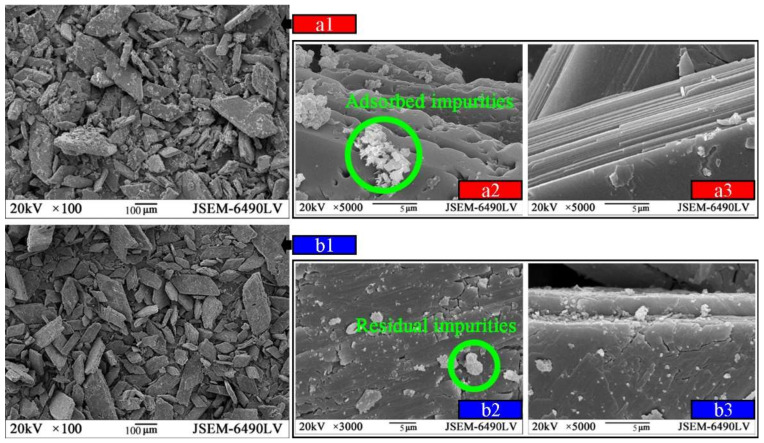
The microstructures of the OPG (**a1**–**a3**) and TPG (**b1**–**b3**).

Table 2 shows that CaO, SO_3_, SiO_2,_ and Al_2_O_3_ were the main oxides both in OPG and TPG, despite the relative content of silica and calcium being raised after water-washings. Phosphate and fluoride, which can negatively affect the mechanical strength and hydration process of CPB samples [11,12,20], both decreased extensively from 1.45% to 0.57% and from 0.93% to 0.42%. The particle size distributions (PSD) of OPG and TPG are plotted in Figure 2. The < 20 μm and < 200 μm particles account for 18.39% and 97.01% of OPG, which are 15.14% and 98.78% for TPG. The microstructures of OPG and TPG were as demonstrated in Figure 3a1,b1. Although the crystal grains of TPG remain rhombic plates, many adsorbed impurities were removed (Figure 3b2,b3).

### 3.2. Rheological Characterization of OCPB and TCPB

**Figure 4:**
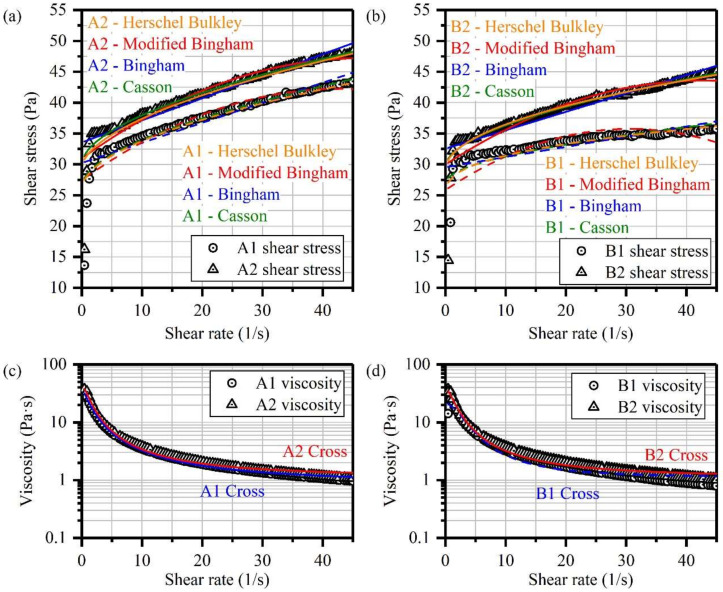
Shear stress and viscosity values of tested mixtures. (**a**) experimental and simulated shear stress curved of A1 and A2 samples; (**b**) experimental and simulated shear stress curved of B1 and B2 samples; (**c**) viscosity values of A1 and A2 samples; (**d**) viscosity values of B1 and B2 samples.

Recorded shear stress and viscosity performing as functions of shear rate are shown in Figure 4. Similar to reference CPB, all the investigated specimens show obvious shear-thinning behaviors and pseudoplastic characters [46,49]. However, with the increase of shear rate, TCPB show more pronounced shear stress. To quantitatively describe the optimization, four rheological models were used as shown in Table S1. The two-parameter Bingham model is commonly used to represent cementitious materials [50]. A second-order term constant *c* is applied in the modified Bingham model to reduce the error at both low and high shear rate conditions [51]. Considering mono-linear models that cannot satisfactorily account for the shear-thinning characteristics of non-Newtonian fluids, the Herschel–Bulkley model and Casson model are also presented [52,53]. The statistical parameter error was calculated as Equation (1) [54]. The Cross model was introduced to characterize the asymptotic viscosities at zero and infinite shear rates. The results determined from the abovementioned models are illustrated in Figure 4 and Table S1.

(1) $${S_{D}=[\frac{\sum_{i=1}^{NDP}{{(X}_{m}-X_{f})}^{2}}{NDP-2}]}^{\frac{1}{2}}$$

where *S_D_* is the statistical parameter error, *X_m_* is the measured value, *X_f_* is the calculated value from each model, and *NDP* is the number of data points.

If only considering the *R* square figures, all the given models show adequate fit responses. However, in terms of the *S_D_* value, the Herschel–Bulkley model performs a more significant fitting result to the tested pastes. Besides, the *τ*_0_ values calculated from the models all demonstrate the optimization of water-washing pre-treatment to yield stress, regardless of the mix proportion. Especially in the Herschel–Bulkley model, *τ*_0_ values of mix proportions A and B are increased by 13.0% and 15.6%. Although *n* values of the tested pastes are all below one, the values of OCPB pastes are smaller than that of TCPB, indicating a more pronounced non-Newtonian characteristic. From the Cross model, the presented *η_∞_* results indicate that the infinite shear viscosity was relatively constant. However, the *η*_0_ values found could be predominant with the OPC concentration and the water-washings. That is to say, although all the flocculant network structures of the tested mortars are all destroyed rapidly after mixing, the microscopic structure of TCPB mortar is more stable than OCPB. In general, the shear-thinning behavior of CPB might be attributed to attractive and repulsive forces, such as Brownian forces and steric hindrance forces [55,56]. After water-washings, the finer-grained TCPB particles can effectively reduce the average distance between particles, and thus particle interaction and shear stress are improved by this micro-filling effect [51,57]. Simultaneously, the improvement might be due to the removal of soluble fluoride and phosphorus, which can significantly retard the early hydration of CPB [11,22]. After experiencing continuous shear mixing, more clusters were generated in TCPB samples where the water was entrapped into the assemblages. However, this could not be confirmed in this experiment but is to consider in further studies.

### 3.3. Mechanical Performance of OCPB and TCPB

**Figure 5:**
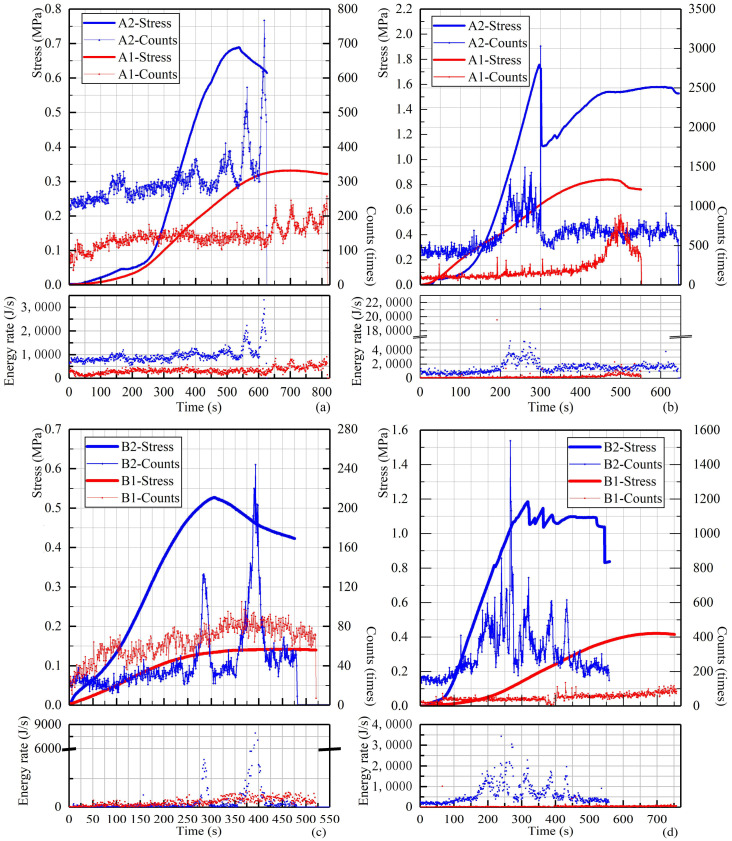
Relation diagrams of stress, AE counts, and energy rate of OCPB and TCPB with 7 days (**a**,**c**) and 28 days of curing (**b**,**d**).

The results present in Figure 5 are the stress curves (left *y*-axis), AE scatterplots (right *y*-axis), and energy rates of OCPB and TCPB specimens. In general, with the water-washing pre-treatment, the unconfined compressive stress values of TCPB samples were significantly enhanced, especially the value (1.19 MPa) of the 28 days cured B2 sample (Figure 5d), which was increased approximately three times higher than the B1 sample (0.42 MPa) although the failure characteristics of the samples are similar. All the counts and energy rate scatters show a positive relation to the loading stress, roughly similar to typical brittle-rigid rocks, which can be classified into four distinctive stages [40]. However, both in the OCPB and TCPB systems, only three stages can be found, quiet compaction, the rapid propagation period, and the macro-fracture initiation period. The beginning period could be related to microcrack contact and slip and pore closure [43]. Then, rapid propagation and macro-fracture initiation appeared, with the AE counts and energy rates gradually increasing and reaching a peak. Besides, it could be found that the AE energy rate and counts increase with the curing ages and OPC content, which means that the generation of hydration products would increase the energy-storing of OCPB and TCPB.

Furthermore, it is evident that the related AE counts of 7 day OCPB samples were more active at the initial period of the UCS test compared with the 28 day samples, if only considering the percentage increase between the initial stage and the peak. This could be related to the pores, mainly consisting of liquid or gas phases, which are presented massively in 7 day OCPB samples. As a result, these pores were rapidly compacted during the initial compression stage, accompanied by large quantities of AE events [58,59]. With the increase of curing time, the pores in the specimen were filled with hydration products [60], and more energy was stored. Therefore, the AE events of 28 day OCPB samples in the initial period are less fluctuant.

Besides, the A2-7D and B2-7D samples showed obvious increase of AE counts after the maximum stress value point. In contrast, the counts–time curves of OCPB perform unimodal distributions. This dislocation of maximum AE counts is usually performed in the compression tests equipped with softer loading platens, for instance, platens with a Teflon thin layer [61]. During the compression tests, friction between loading platens and the specimen was prone to produce material fragmentation near the bases [62]. When using the soft loading platens, outward-directed shear forces would generate at the interface and then the associated lateral deformation entailed the failure from crushing to splitting. Whereas, as presented in previous studies [62,63], platen restriction also shows side effects on AE events before the maximum stress is accompanied by more apparent AE signals in the post-peak period. Previous studies supported that the dislocation of maximum AE counts could presumably be affected by the cementation effect [64]. Thus, accompanied by the platen restriction and the OPC content, water-washings can also activate AE events in the post-peak period.

#### 3.3.1. Internal Damage Fractal Dimension (D) Characteristics

The fractal dimension *D* is any dimension measurement that allows non-integer values, and the fractal is the set of *D* [42,59]. As a widely used method for estimating fractal dimensions of experimental data sets, the complexity of the internal damage evolution of the specimen can be expressed by *D* values. Meanwhile, the Grassberger–Procaccia (G–P) algorithm [65] is widely used to estimate *D* values of data sets that include a time sequence. Once the set of points (ln *r*, ln *C*(*r*)) demonstrates a linear correlation relationship in a log–log coordinate, it can be concluded that the fitted set has fractal characteristics at a given scale. Hence, the fractal dimension *D* can be estimated as the slope of the straight-line portion

ln *C*(*r*) = D*ln *r* + *d* (2)

where *d* is the coefficient constant representing the material characteristic. Thus *C*(*r*) is approximately given by Equation (3)

(3) $$C(r)\approx \frac{1}{N^{2}}\sum_{i=1}^{N}\sum_{j=1}^{N}{H(r-|x}_{i}{-x}_{j}\left|\right)$$

where *N* = *n* – *m* + 1, *n* is the dimension of the original constructed phases space, after *m* (*m* < *n*) isolated sets of points have been removed from the constructed space, *N* vectors can be obtained from reconstructed phases space, and |*x_i_*−*x_j_*| is the distance between any pair of points. The Heaviside function [66] is defined by Equation (4)

(4) $$H\left(s\right)=\left\{\begin{array}{c} 1,\;\mathrm{if}\;s\;\geq \;0\\ 0,\;\mathrm{if}\;s\;<\;0 \end{array}\right.$$

The value of *r* can be calculated by Equation (5)

(5) $$r=s\times \frac{1}{N^{2}}\sum_{i=1}^{N}\sum_{j=1}^{N}{|x}_{i}-x_{j}|$$

where *s* is the scale factor, to acquire a prominent fractal characteristic, the *s* values are given as 0.2, 0.4, 0.6, 0.8, 1.0, 1.2, and 1.4. While using the G–P algorithm, a previous study [66] demonstrated that an appropriate reconstructed phase space needs to be determined to promote the accuracy of the fractal analysis. Therefore, a new parameter of *D_m_* was defined as the ratio of ln *C*(*r*) to ln *r*. Then, the *m* value was selected in the period when the *D_m_* values gradually kept stabilizing. Figure S2 plots the change of *D_m_* with m values ranging from 2 to 30.

**Figure 6:**
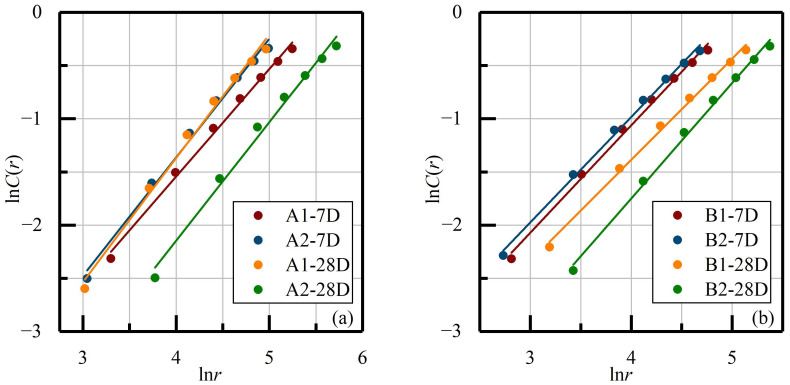
The relationship between phase space and correlation dimensions for the AE signals during monotonic compression. (**a**) samples with proportion A; (**b**) samples with proportion B.

It was shown clearly that, apart from the A2-7D and B2-28D samples, the computed *m*-*D_m_* curves’ general trend all remain steady, especially the *D_m_* values of OCPB specimens which stabilize from 0.90 to 0.95. After a short period of increase, the *D_m_* values of A2-7D and B2-28D samples re-stabilizes at *m* values at 11 and 20. Therefore, the figure for the phase space dimension *m* was chosen as 20 for the AE fractal analysis for the further step. In addition, the values of TCPB were concentrated at ranges from 0.98 to 1.05 and from 1.15 to 1.80, respectively, which are far higher than OCPB, indicating that besides the contribution of OPC content and the curing age, water-washing pre-treatments also greatly enhance the fitted *D_m_* values. Subsequently, with the determined m values, the ln *C*(*r*) and ln *r* values were plotted as in Figure 6, and the parameters of the fitted straight lines introduced as in Table 3.

From the calculated *R*^2^ figures, it can be seen that all the ln *C*(*r*) and ln *r* values demonstrate obvious linear correlation, proving that the tested specimens all have fractal characteristics. For the A1 and A2 samples, the *D* values slightly increased with the curing age, which is not observed in ratio B. Besides, although different proportions were prepared, the *D* values remain approximately constant. The possible factors which correlate to this behavior, with the *D* values fluctuating around 1, could be ascribed to the inherent character of weak strength and residual stress after loading.

**Figure 7:**
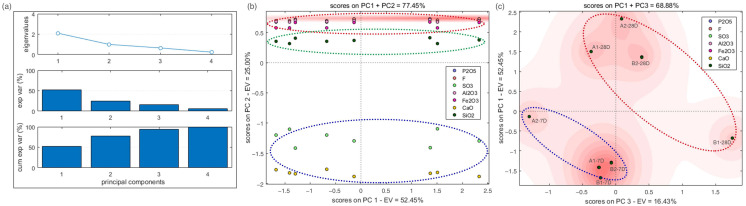
PCA for the influence of curing ages and chemical composition on mechanical properties and *D*-values. (**a**) Elbow plot of PCA. (**b**) PCA with principal components 1 and 2 (**c**) PCA with principal components 2 and 3.

In order to limit the variables in the experimentation and obtain a correlation between the previous dependent and independent variables, the PCA results of the D-values, curing ages, and UCS values are collated in Figure 7 [67,68]. From the scree plot in Figure 7a, the first three eigenvalues represent approximately 94% of the total variabilities indicating that the PCA results are reasonable. In Figure 7b,c, the PCA bi-plots of two principal components reflect the relationship among the variable bulk chemical data, curing ages, and mechanical performance. In the axis of principal component 2, the impact of fluorine and phosphorus were preceded only by calcium and sulfate, which are the primary ions in OPG and TPG. According to Figure 7c, the contribution of curing ages was shown by diagonally classifying the studied specimens into two groups.

#### 3.3.2. Loading Time Sequence Investigation: *D_N_*-Values and *b*-Values Analyses

**Figure 8:**
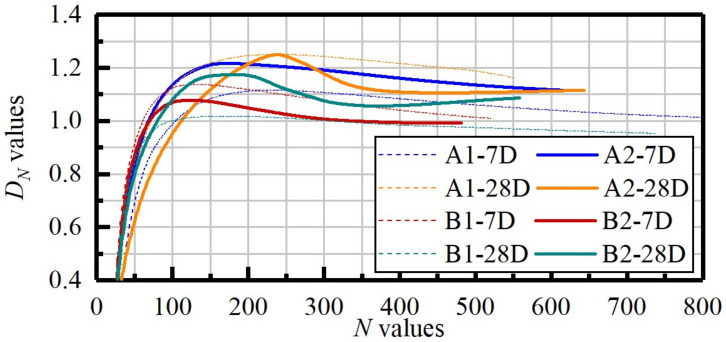
The relationship between *D_N_*-*N* during monotonic compression.

Since the opting for *N* values is based on the time used for a completed compression process, *D_N_* is assumed to identify the energy released per second of modalities in each structural element. The calculated *D_N_* values are summarized in Figure 8. Besides, being a significant parameter for monitoring and forewarning impending failure in engineering materials, the *b*-value is derived from the seismic magnitude–frequency equation (Equation (6)) [59]. It represents the scaling of magnitude distribution of AE signals, showing temporal fluctuations as the impending failure approaches in the material. Usually, a high b-value reflects an overwhelming number of small AE events generated, representing new crack formation and slow crack growth. In contrast, a low *b*-value indicates faster and unstable crack growth accompanied by relatively high amplitude AE events in large numbers.

log *H* = *a* − *b* × log *A* (6)

where *H* is the number of hits having amplitudes larger than *A*, *A* is the signal amplitude (dB), *a* is an empirically derived constant, 2.5 is used in this study.

**Figure 9:**
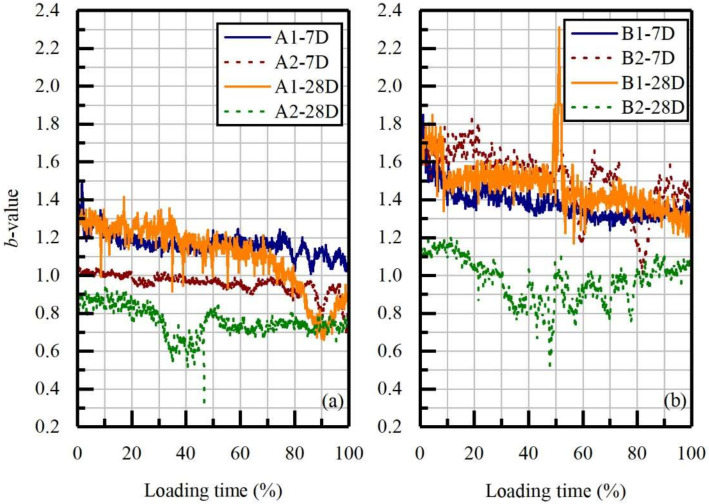
*b*-values of tested specimens. (**a**) samples with proportion A; (**b**) samples with proportion B.

By defining *D_N_* values as the function of loading time, more delicate information was captured. At the initial stage of uniaxial loading, all *D_N_* values exhibited a linear upward trend corresponding to the coalescence of pre-existing voids and the rubbing of cracks [69]. Because of the different bearing capacities of the anisotropic samples, the fractal dimension successively increased to the maximum values. Then, as the microcracks gradually converged to specific major crack zones with the AE events showing more order distribution, they then respectively decreased to minimums, ranging from 0.8 to 1.1. However, at the end of loading, the A2-28D and B2-28D samples showed a rebound trend which could be explained by the squeezing and collision of the residual structures. The *b*-value results (Figure 9) show that all the tested samples decreased gradually during the initial stage, which corroborated the investigations of *D_N_* values in the initial stage. Besides, samples with higher OPC content and water-washing pre-treatment showed more substantial fluctuations representing the release of stored energy. The downward trend was demonstrated in the final loading stage for the 7 day cured samples with A1 and A2 proportions (Figure 9a). However, in B1-7D and B2-7D, an intertwined trend was revealed instead of downward, which could be related to the low OPC ratio. Besides, curing can bring a downward trend to the *b*-values, especially in the water-washing treated samples. To progressively understand the relevance between AE signals and hydration systems, the mineralogical composition and morphology were studied.

### 3.4. Microstructure Analysis

**Figure 10:**
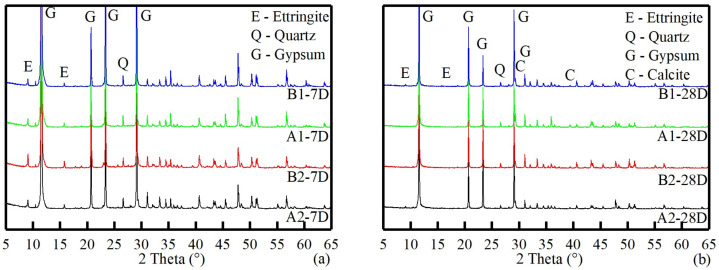
XRD patterns of OCPB and TCPB samples in 7 days (**a**) and 28 days (**b**).

X-ray diffraction patterns of OCPB and TCPB samples are presented in Figure 10. It can be seen that the main crystalline hydration product at 7 days was ettringite. At 28 days, the intensity of gypsum significantly decreased, but there was still a large amount of unreacted gypsum present, while the ettringite partially carbonized and produced calcite. In general, carbonization at early hydration can enhance concrete resistance to sulfate attack, chloride penetration, and water absorption [70] but could bring severe threats to concrete structures [71]. In this study, no reduction of compressive strength was observed. Despite attempts to reduce the exposure to atmospheric CO_2_, it was confirmed that it can decompose by carbonation at 25 °C in a moist atmosphere [31,72]. As regards the source of quartz, this might be attributed to the raw materials instead of the decomposition of CSH [73] because the diffraction peaks in 28 days were not significantly enhanced.

**Figure 11:**
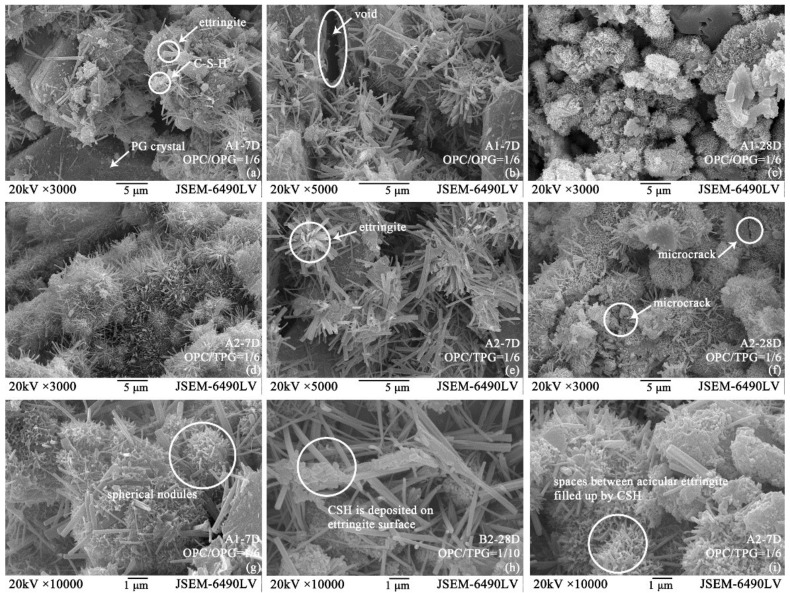
SEM images of OCPB and TCPB samples. (**a**,**b**) Internal microstructure of 7 days cured A1 sample; (**c**) internal microstructure showing voids still within the 28 days cured A1 sample; (**d**,**e**) 7 days cured A2 sample; (**f**) 28 days cured A2 sample demonstrating a more compact structure; (**g**–**i**) amorphous hydrates filled the internal cracks.

The morphology shows that despite some micro-cracks and uncovered gypsum crystals being observed after 28 days of curing, the initial water-filled space reduced significantly, comparing the 7 day open and friable microstructures (Figure 11a,c,d,f). Similar to the hydrated OPG-based cement system [1,18], hydration products gradually bond unreacted particles to form a solid binder with the formation of an advanced hydration stage. The ettringite generates between the gypsum particles with amorphous hydrate evolving into the structure, filling the initial water-filled space (Figure 11g,h). Moreover, most of the hydration product images in Figure 11 are dominated by large acicular crystals, typically of the order of 0.2~0.5 μm in width and ranging from about 2 μm to over 10 μm in length but little growth occurred subsequently. The gathered CSH gel alternatively filled up the spaces between the acicular ettringite and spherical nodules formed (Figure 11g,i). Besides, even in 28 days, ettringite and CSH gel were the main hydration products instead of monosulfoaluminate and portlandite, which was also verified by XRD. That is because the excess sulfate system and acidic source can induce ettringite formation and portlandite consumption [74]. Furthermore, as demonstrated in the XRD tests, each sample gradually carbonized at the middle and late stages, and a large number of products such as quartz and calcite appeared [31,73]. Hence, after carbonization, precipitation of crystals occupied the spaces of pores and voids, identified as the weakest links.

**Figure 12:**
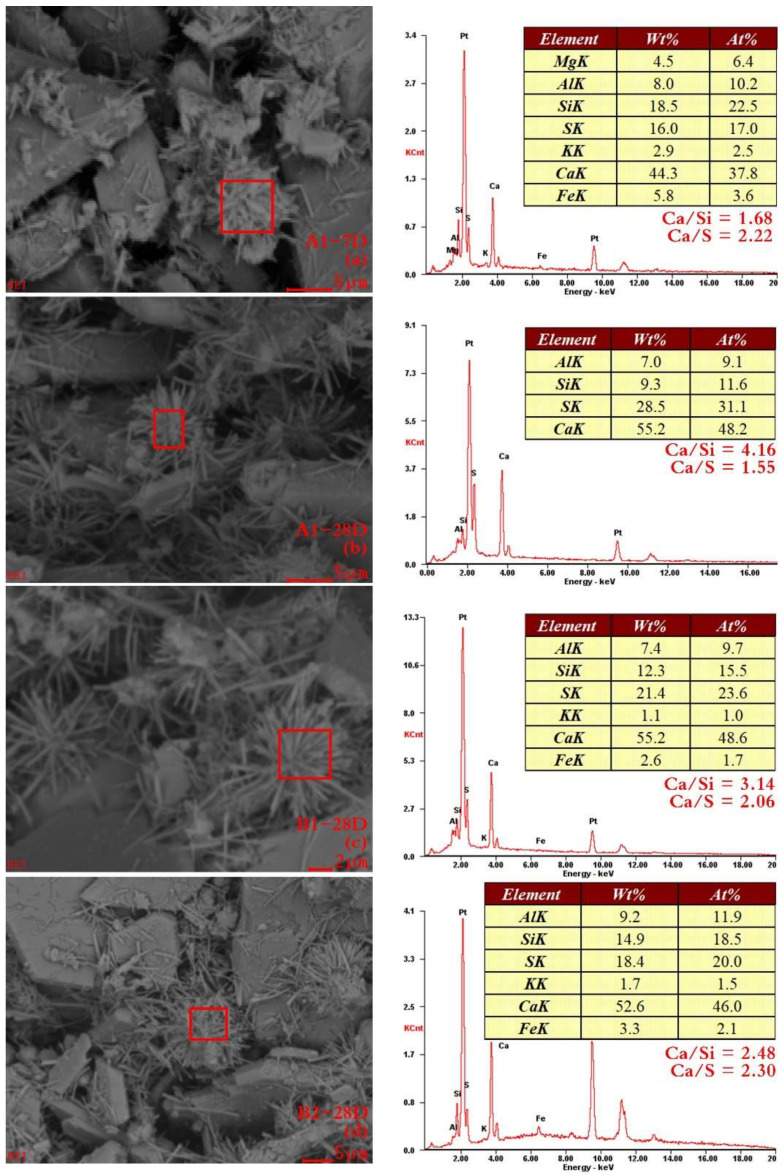
SEM/EDX images of OCPB and TCPB samples. (**a**,**b**) 7 and 28 days cured A1 samples; (**c**,**d**) 28 days cured B1 and B2 samples.

Although many studies have been conducted [70,71,73], the relation between the chemical composition of hydration products and the macroscopic mechanical properties of the hardened material is not well understood. However, an ettringite assembly of stellate clusters (Figure 11e) has been confirmed that would have a significant contribution to the mechanical properties due to the interlocking of the “arms” from adjacent clusters [75]. Moreover, the presence of the stellate clusters seems to validate the ettringite formation and expansion mechanism [76,77,78]. Figure 12 presents the EDS spectra from stellate ettringite. Several regions were analyzed with EDS and were found to have similar compositions regardless of whether or not they had had water-washing pre-treatment. However, this can be used to eliminate the possibility that the observed phases were other hydrates with similar morphologies. The calculated Ca/S atomic ratios (Figure 12a,c,d) were from 2.06 to 2.30, which are close enough to the theoretical ratio of 2 for ettringite. In contrast, the calculated ratio of the A1-28D sample was only 1.55. It could be responsible for the non-ideal EDS specimens. The uneven flat cannot provide accurate quantitative data [75]. Therefore, the presence of calcite, which presents as the core of the stellate cluster, could be involved because a high Ca/Si ratio was observed.

## 4. Conclusions

The main goal of this study was to quantify the rheological characterization and failure performance of TCPB, thereby supplying more information for OPG recycling scenarios. Based on the results and discussion, the following conclusions were drawn:

In rheological characterization, all tested pastes demonstrated great shear-thinning and pseudoplastic characters, and the generalized Herschel–Bulkley model had the lowest deviation value. Besides, after water-washings, 13% and 16% of yield stress increase were observed with the ratios of A and B, respectively. The optimized particle size distribution of TPG could contribute to the non-Newtonian properties of TCPB mixtures.

Accompanied by more active AE events, the mechanical properties of the TCPB significantly increased. In both systems, the deformation performance and the activity of AE signals during compression were positively correlated. The AE counts occur during the quiet compaction period, whereas fragmentation dominates in the later stages. Two systems demonstrated similar internal damage fractal characteristics, with the fractal dimensions decreased to approximately 1.0 at the major crack generation period. With PCA, fluorine and phosphorus were confirmed to have an essential side effect on the fractal dimensions, preceded only by calcium and sulfate.

By defining fractal dimension values as a function of loading time, TCPB showed a rebound trend at the final stage, indicating more robust residual structures in these uniaxially compressed specimens. In addition, water-washings can bring downward trends to the *b*-values, representing unstable cracks with a high amplitude which were prone to generate in TCPB samples.

Through the microstructure and mineralogical analysis, water-washings obviously optimized the pore structures. Another interesting observation is that, in both systems, acicular ettringite was found that joins in the form of stellate clusters. Such a radiative structure may produce better mechanical properties, with the stress to break the interlocked “arms” contributing to the residual stress and the *b*-values rebound trend in TCPB. However, the hardened phase assemblage hypothesis is the more likely explanation and needs to be developed for future progress.

## Figures and Tables

**Table 1:** Mix proportions of the OCPB and TCPB samples.

No.	OPG (Dry, wt%)	TPG (Dry, wt%)	Water (wt%)	OPC (wt%)	OPC/Water	OPG (TPG)/OPC
A1	51.43	-	40.00	8.57	0.214	6:1
B1	54.55	-	40.00	5.45	0.136	10:1
A2	-	51.43	40.00	8.57	0.214	6:1
B2	-	54.55	40.00	5.45	0.136	10:1

**Table 2:** The chemical composition of OPC, OPG, and TPG.

Chemical Composition (%)	OPC	OPG	TPG
Na_2_O	0.10	0.14	0.06
MgO	1.82	0.11	0.01
Al_2_O_3_	4.32	0.80	0.29
SiO_2_	17.85	6.24	5.01
P_2_O_5_	0.21	1.45	0.57
SO_3_	3.49	40.02	44.43
Cl	0.02	-	-
K_2_O	0.66	0.62	0.31
CaO	61.02	45.80	47.15
TiO_2_	0.30	0.69	0.55
V_2_O_5_	0.04	-	-
Cr_2_O_3_	0.03	-	-
MnO	0.27	-	-
Fe_2_O_3_	3.80	3.01	1.08
CuO	0.01	-	-
ZnO	0.04	-	-
SrO	0.07	0.10	0.06
ZrO_2_	0.01	-	-
BaO	0.04	0.08	0.07
F	-	0.93	0.42
Loss	5.91	-	-

**Table 3:** Fitting parameters of ln *C*(*r*) − ln *r*.

Samples	*D*	d	R^2^	Samples	*D*	d	R^2^
A1-7D	1.011	−5.589	0.995	B1-7D	1.009	−5.100	0.996
A2-7D	1.115	−5.821	0.994	B2-7D	0.993	−4.956	0.997
A1-28D	1.162	−6.019	0.992	B1-28D	0.953	−5.199	0.996
A2-28D	1.116	−6.611	0.990	B2-28D	1.087	−6.097	0.996

## Data Availability

The data presented in this study are available on request from the corresponding author.

## Supplementary Materials

The following are available online at https://www.mdpi.com/article/10.3390/ma14226993/s1, Figure S1: Diagram of UCS tests accompanied with AE system, Figure S2: Change in *D_m_* values depending on *m* values selection, Table S1: Calculated parameters of the rheological models.

## Abbreviations

AE	Acoustic emission
CPB	Cemented paste backfill
CSH	Calcium silicate hydrates
DW	Deionized water
OCPB	Ordinary phosphogypsum based cemented paste backfill
OPC	Ordinary Portland cement
OPG	Ordinary phosphogypsum
PCA	Principal component analysis
PSD	Particle size distribution
SEM	Scanning electron microscopy
TCPB	Water-washing treated phosphogypsum based cemented paste backfill
TPG	Water-washing pre-treated phosphogypsum
UCS	Uniaxial compression strength
XRD	X-ray powder diffraction
XRF	X-ray fluorescence
