# Supplementary material for: In Situ Remediation of Phosphogypsum with Water-Washing Pre-Treatment Using Cemented Paste Backfill: Rheology Behavior and Damage Evolution
Source: Materials (Basel). 2021 Nov 18;14(22):6993. doi: 10.3390/ma14226993 (PMC8618653; doi:10.3390/ma14226993)
Supplement: Supplementary file 1 [file materials-14-06993-s001.zip › materials-1424079-supplementary.pdf]

materials

Supplementary Files

In Situ Remediation of Phosphogypsum with Water-Washing Pre-Treatment Using Cemented Paste Backfill: Rheology Behavior and Damage Evolution

Yikai Liu [1,3], Qiusong Chen [2,3,*], Yunmin Wang [2,3], Qinli Zhang [3], Hongpeng Li [4],Chaoyu Jiang [3], and Chongchong Qi [3]

1 Department of Geosciences, University of Padova, 35131 Padova, Italy; yikai.liu@phd.unipd.it
2 School of Resources and Safety Engineering, Central South University, Changsha 410083, China; Wangyunmin@csu.edu.cn (Y.W.); zhangqinlicn@126.com (Q.Z.); 13278883353@163.com (C.J.); chongchong.qi@gamil.com (C.Q.)
3 Sinosteel Maanshan General Institute of Mining Research Co., Ltd., Maanshan 243000, China
4 Yinshan Mining Co., Ltd., Jiangxi Copper Group, Dexing 334200, China; lihongpeng88@126.com
* Correspondence: qiusong.chen@csu.edu.cn; Tel.: +86-0731-8887-9612

Table S1. Calculated parameters of the rheological models.

Rheological models		Mix proportions			
**Model name**	**Parameters**	**A1**	**A2**	**B1**	**B2**
Bingham ($\tau = \tau_0 + \mu_p \cdot \dot{\gamma}$)	τ_0	30.230	33.555	29.544	32.547
	μ_p	0.325	0.357	0.165	0.298
	R^2	0.988	0.991	0.971	0.989
	S_D	2.018	1.965	2.682	2.033
Modified Bingham ($\tau = \tau_0 + \mu_p \cdot \dot{\gamma} + c \cdot \dot{\gamma}^2$)	τ_0	27.486	30.958	25.848	29.958
	μ_p	0.693	0.700	0.648	0.639
	c	-0.008	-0.008	-0.011	-0.007
	R^2	0.993	0.995	0.982	0.994
	S_D	1.746	1.814	2.537	1.915
Casson ($\tau^{0.5} = \tau_0^{0.5} + \mu_p^{0.5} \cdot \dot{\gamma}^{0.5}$)	τ_0	26.259	29.771	28.578	29.497
	μ_p	0.050	0.049	0.010	0.035
	R^2	0.991	0.993	0.994	0.993
	S_D	1.580	1.611	2.528	1.727
Herschel–Bulkley ($\tau = \tau_0 + k \cdot \dot{\gamma}^n$)	τ_0	25.975	29.354	25.026	28.934
	k	2.323	2.225	2.728	2.472
	n	0.527	0.558	0.370	0.483
	R^2	0.993	0.994	0.994	0.970
	S_D	1.507	1.572	2.405	1.702
Cross ($\mu_v = \eta_\infty + (\eta_0 - \eta_\infty) / [1 + (t \cdot \dot{\gamma})^m]$)	η_0	37.469	43.920	21.009	38.524
	η_∞	0.988	1.096	1.099	1.091
	t	0.661	0.652	0.318	0.573
	m	1.402	1.521	2.099	1.604
	R^2	0.998	0.996	0.936	0.993

*where τ_0 means the yield stress (Pa), μ_P means the plastic viscosity (Pa·s), $\dot{\gamma}$ is the shear rate (1/s), c and k are constants, n is the flow index, μ_v represents the viscosity, η_0 and η_∞ are the zero-shear viscosity and infinite shear viscosity, t is constant, and m is the power instant.

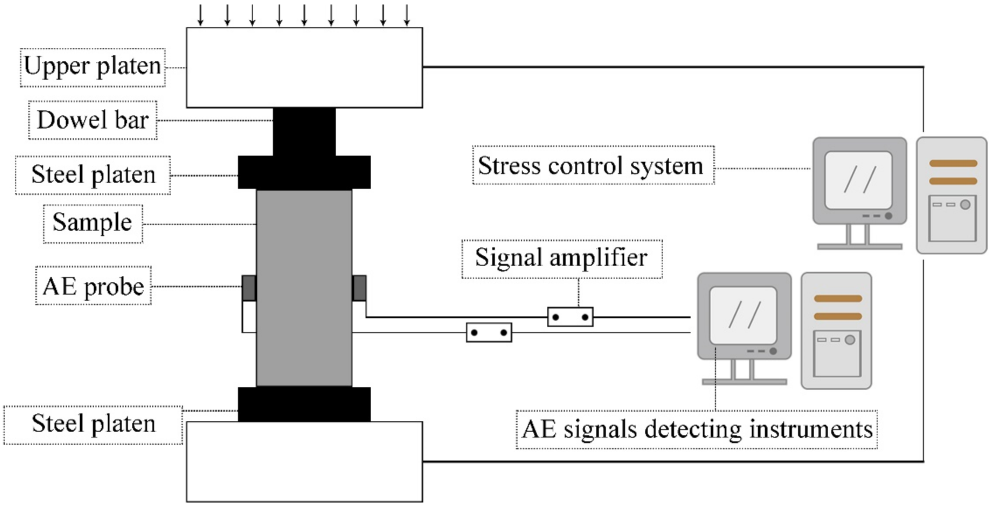

Figure S1. Diagram of UCS tests accompanied with AE system.

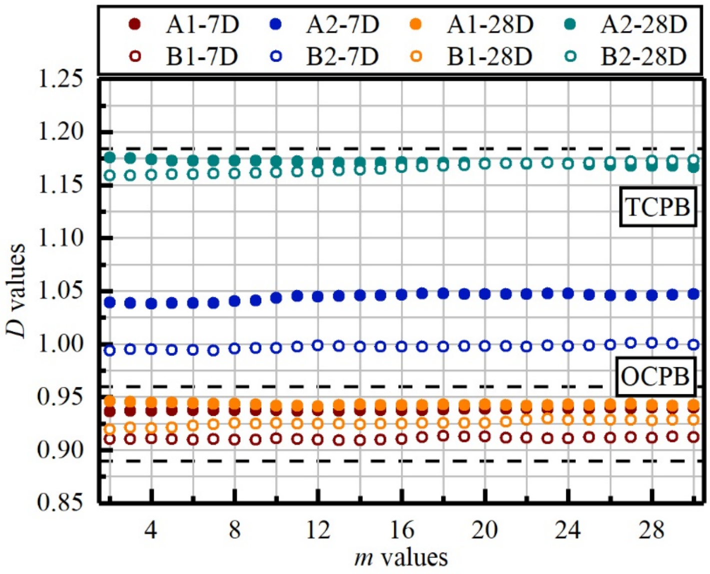

Figure S2. Change in D_m values depending on m values selection.

© 2021 by the authors. Submitted for possible open access publication under the terms and conditions of the Creative Commons Attribution (CC BY) license (http://creativecommons.org/licenses/by/4.0/).
